# Colonic atresia and Hirschsprung's disease in a neonate: A case report

**DOI:** 10.1016/j.ijscr.2024.110250

**Published:** 2024-09-07

**Authors:** Thomas P. Schermoly, Kurt P. Schropp

**Affiliations:** Department of Surgery, University of Kansas Medical Center, Kansas City, KS 66103, USA

**Keywords:** Colonic atresia, Hirschsprung's, Neonate, Case report

## Abstract

**Introduction:**

Colonic atresia is a rare form of intestinal atresia that can be encountered in neonates. Although uncommon, other disease processes can be found simultaneously including malrotation, additional atresias, gastroschisis, and Hirschsprung's disease.

**Case presentation:**

A 2-day-old female neonate with known maternal polysubstance use was found to have colonic atresia on contrast enema after emesis and failure to pass meconium. Abdominal exploration revealed a blind ending cecum with evidence of ischemia along with an atretic transverse colon. An ileocecectomy with end ileostomy and transverse colon mucous fistula creation were performed. After eventual ileostomy reversal at 5 weeks of age, she struggled with intermittent oral intolerance and inconsistent bowel function. *Re*-exploration with ileostomy and gastrostomy tube placement was performed with additional biopsies revealing Hirschsprung's disease.

**Clinical discussion:**

Concomitant colonic atresia and Hirschsprung's disease is a rare clinical entity that provides challenges in diagnosis and definitive surgical management. The suspected source of atresia in this case was presumed to be due to an intra-uterine vascular accident given maternal polysubstance use. Delays in diagnosis can lead to increased patient morbidity.

**Conclusion:**

Even with a clear suspected etiology for colonic atresia, surgeons must maintain a high clinical suspicion for additional pathologies including but not limited to Hirschsprung's disease. Rectal suction biopsies should be performed if clinical suspicion arises for Hirschsprung's disease.

## Highlights

• Colonic atresia with concomitant Hirschsprung's disease is a challenging clinical entity.
• Deviation in clinical course should prompt repeat rectal suction biopsy.
• Rectosigmoid ratio and malfixed colonic segments are not reliable for diagnosis in all cases of Hirschsprung's disease.

## 1 Introduction

Colonic atresia is a rare entity, accounting for only 2–15 % of intestinal atresias overall [1]. Estimates of incidence are approximately 1 in 20,000 live births. Diagnostic workup and therapeutic intervention are time sensitive as delay in intervention beyond 72 h has shown to significantly increase risk of mortality [2]. However, anomalies may be complex and can be associated with other pathology including additional intestinal atretic segments, malrotation, gastroschisis, and Hirschsprung's disease [3,4]. Complex pathology can complicate surgical management and intra-operative decision-making.

Here we described a patient initially found to have a right-sided colonic atresia, whose prolonged hospital course in an academic medical center under the care of an established pediatric surgeon revealed a concomitant diagnosis of long segment Hirschsprung's disease.

## 2 Case report

**Fig. 1:**
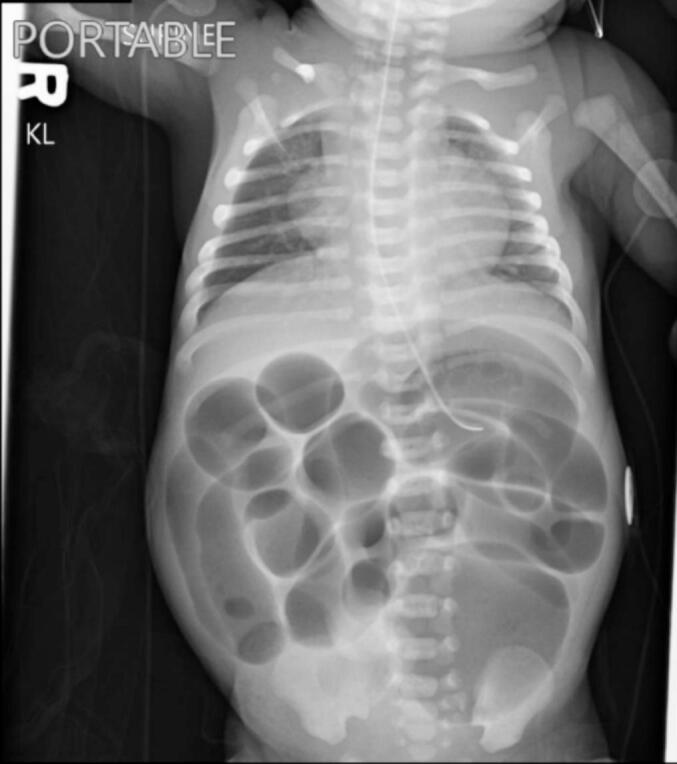
Pre-operative KUB obtained following failure to pass meconium and emesis showed non-specific dilated loops of bowel.

**Fig. 2:**
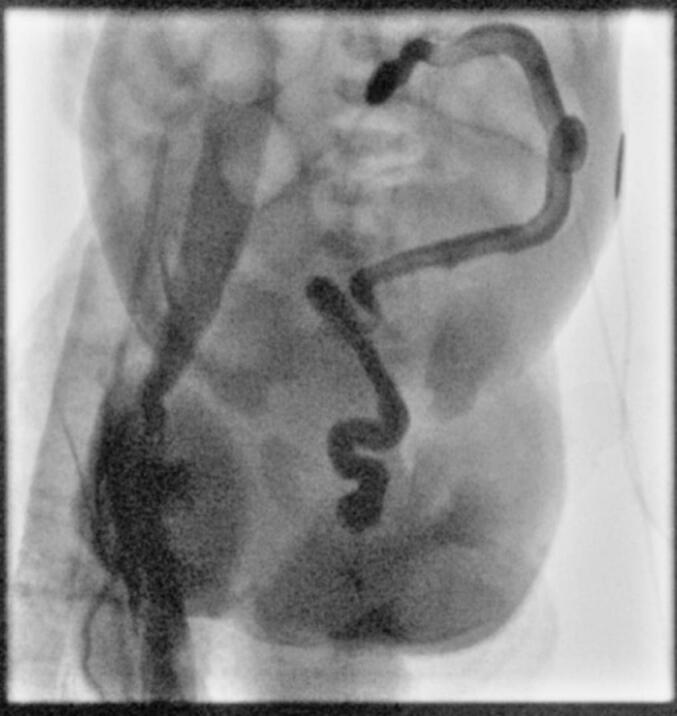
Contrast enema obtained prior to her index operation showed contrast termination in the mid transverse colon.

A 2-day-old female was born at 38 weeks and 1 day with limited prenatal care. She was born via Cesarean section due to breech position and had a nuchal cord that required reduction. Apgars at 1 and 5 min were 7 and 9, respectively. Birth weight was 2205 g. Maternal drug screen was positive for fentanyl and amphetamines along with reported THC and tobacco use. Following birth she was monitored in the neonatal intensive care unit (NICU) for respiratory distress before stabilizing and being transferred to the floor. Poor oral intake, emesis of formula feeds, and failure to pass meconium prompted transfer back to the NICU along with surgical consultation. She had developed abdominal distension, prompting Replogle placement with improvement. A KUB was obtained with dilated loops of bowel but no evidence of pneumoperitoneum, as seen in Fig. 1. A temperature probe could be passed rectally and broad-spectrum antibiotics were initiated. A contrast enema was then obtained with decreased caliber of the rectum, sigmoid, descending, and transverse colon with abrupt termination of contrast in the mid transverse colon, shown in Fig. 2.

Abdominal exploration revealed a significantly dilated and ischemic appearing cecum along with an atretic transverse colon and completely absent right colon. An ileocecectomy was performed with end ileostomy and transverse mucous fistula creation. Biopsies of the atretic colon were performed. Pathology showed evidence of narrowing and mild fibrosis, but ganglion cells were present. She was provided nutritional support with total parenteral nutrition (TPN) and enteral feeds, and retrograde saline enemas to assist with colonic maturation. After repeat contrast enema showed no evidence of colonic stricture, she returned to the operating room at 5 weeks of age for ostomy reversal and an ileocolonic handsewn anastomosis, with a notable ileocolonic size mismatch. Following inconsistent bowel function and oral feeding intolerance, rectal suction biopsies were performed showing absent ganglion cells, consistent with Hirschsprung's disease. At 10 weeks of age, she was taken for end ileostomy creation along with repeat colonic biopsies and gastrostomy tube placement. Final pathology showed absent ganglion cells in the colonic tissue at the ileocolonic anastomosis and distal colon, consistent with long segment Hirschsprung's disease. This delay in diagnosis ultimately resulted in a Clavien-Dindo classication 3b for intervention under general anesthesia.

She remained inpatient for ongoing nutritional support, monitoring of ileostomy output, and appropriate growth until stable discharge home at four months of age.

This work has been reported in line with the consensus surgical case report (SCARE) criteria [5].

## 3 Discussion/conclusions

Colonic atresia, although a rare entity in isolation, can present a significant dilemma for clinicians and surgeons. As mentioned previously, prompt diagnosis of colonic atresia, which often leads to proximal obstruction, can have significant affects on morbidity and mortality [2]. Further complicating care is the presence of concomitant pathology such as Hirschsprung's disease – which is often diagnosed in a delayed fashion. One review showed average age at diagnosis of Hirschsprung's disease in patient's also found to have colonic atresia to be 8 months [6]. Due to the delay in diagnosis the most commonly cited reason for discovering Hirschsprung's disease has been clinical deterioration in the form of anastomotic breakdown following a previous ileo-colonic or colo-colonic anastomosis [7]. The potential for anastomotic leak, obstruction, or sepsis places these patients at significant risk of post-operative complications and need for re-operation. Factors predicating the presence of Hirschsprung's disease in these patients include abnormal or improper fixation of the colon distal to the atretic segment [8]. Recommendations include biopsy of any abnormal or “malfixed” colonic segment at the time of surgery.

Our patient presented a unique diagnostic challenge as 1) maternal polysubstance use provided a known etiology for her colonic atresia and 2) biopsies of the distal colonic segment at the index operation showed the presence of ganglion cells. She had normal fixation of the colonic segment distal to the atresia, suggesting Hirschprung's disease may have been less likely. There was also no additional imaging evidence concerning for Hirschsprung's on contrast enema or plain films. Fortunately, her hospital course was not complicated by clinical instability, but rather a lack of consistent bowel function and expected clinical progress, prompting eventual rectal suction biopsy and a formal diagnosis of Hirschsprung's disease. This was followed by subsequent diverting end ileostomy and gastrostomy tube placement with plans to determine eligibility for a definitive pull through operation at a later date.

Clinical suspicion for associated anomalies with colonic atresia, including Hirschsprung's disease must remain high, particularly in the case of deviation from an anticipated post-operative course. We recommend a low threshold for rectal suction biopsy or repeat biopsy to avoid diagnostic confusion and potential for repeat operations in a population at risk for peri-operative morbidity and mortality.

## Consent

Written informed consent was obtained from the patient's parents/legal guardian for publication and any accompanying images. A copy of the written consent is available for review by the Editor-in-Chief of this journal on request.

## Ethical approval

Ethical approval for this case report was provided by the University of Kansas ethics committee.

## Funding

We declare that no funding was received in support of this work.

## Author contribution

Thomas Schermoly, DO – primary author, corresponding author, and primary contributor.

Kurt Schropp, MD – supporting author/supervising physician.

## Guarantor

Thomas Schermoly, DO.

## Research registration number

1. Name of the registry: N/A.
2. Unique identifying number or registration ID: N/A.
3. Hyperlink to your specific registration (must be publicly accessible and will be checked): N/A.

## Conflict of interest statement

We declare there are no conflicts of interest.
